# Developing a Content Model of a Mobile-Based Application to Manage Patients with Low-Back and Neck Pain

**DOI:** 10.1155/2024/8415777

**Published:** 2024-01-05

**Authors:** Yasaman Farjami Rad, Leila Shahmoradi, Noureddin Nakhostin Ansari, Scott Hasson, Maryam Ebrahimi, Meysam Rahmani Katigari

**Affiliations:** ^1^Sports Medicine Research Center, Neuroscience Institute, Tehran University of Medical Sciences, Tehran, Iran; ^2^Health Professions Education Research Center, Tehran University of Medical Sciences, Tehran, Iran; ^3^Health Information Management Department, School of Allied Medical Sciences, Tehran University of Medical Sciences, Tehran, Iran; ^4^Department of Physiotherapy, School of Rehabilitation, Tehran University of Medical Sciences, Tehran, Iran; ^5^Research Center for War-affected People, Tehran University of Medical Sciences, Tehran, Iran; ^6^Department of Physical Therapy, Augusta University, Augusta, Georgia, USA; ^7^Department of Health Information Technology, Neyshabur University of Medical Sciences, Neyshabur, Iran; ^8^Department of Health Information Technology, Saveh University of Medical Sciences, Saveh, Iran

## Abstract

**Introduction:**

As a complementary tool in health, the design of mobile applications to influence care and increase awareness of patients has grown a lot. The purpose of this study is to design and validate the content model of a mobile-based application for managing patients with low-back and neck pain.

**Methods:**

This descriptive-analytical study was conducted in two main stages to determine the content model of the application. The first stage consisted of three steps: finding the right exercise, determining the right scale to assess the pain intensity, and determining the appropriate features of the application. In the second stage, data elements collected from the previous stage were prepared in the form of a questionnaire that was given to 12 experts in physical therapy and sports medicine for validation. After collecting the questionnaire, data elements in all parts were analyzed based on the content validity ratio (CVR) and descriptive statistics indicators.

**Result:**

The content of the application was prepared in the three axes of exercises for low-back and neck pain, assessment of pain intensity, and features of the application. In the axis of sports exercises, 8 exercises for back pain and 3 exercises for neck pain were included according to the reference books. A Functional Rating Index (FRI) scale with 10 elements was selected in the axis of determining pain intensity. Also, 12 features such as the daily exercise section, using the animation, and using an audio file to explain how to do exercises were included in the model.

**Conclusion:**

According to the gaps identified in the existing applications, determining the content model of the application that is based on evidence and according to the opinion of experts is useful in improving the apps. The content model of this study was presented in 3 axes to increase the patient's willingness to do exercises, the correct way to perform exercises, conservative treatment, and check the progress of the treatment. The software developers can use these findings as a basis for designing new apps to manage low-back pain and neck pain.

## 1. Introduction

In the past, global health priority was on infectious diseases. But with the growth of population, increase in life expectancy, and decrease in mortality, noncommunicable diseases such as musculoskeletal disorders have become common [1, 2]. In this century, according to estimates, the burden of musculoskeletal disorders is very high, and among its common diseases, osteoarthritis, rheumatoid arthritis, gout, low-back pain, and neck pain can be mentioned [3, 4]. In 2019, its age-standardized prevalence was reported as 27.0 per 1000 people [5]. Also, in 2012, 25.5 million Americans were absent from work due to neck pain problems, missing an average of 11.4 days of work [6]. Low-back pain is also a common complaint, especially in industrialized societies, with chronic low-back pain reported in 54.0 to 90.0 percent of the adult population. It is considered a public health problem and is the most common complaint among workers in all fields [7]. These two disorders are common reasons for visiting medical centers and have a significant impact on individuals, communities, healthcare systems, and businesses [8, 9]. In Iran, these two disorders are responsible for 11.4% of all years of life with disability [10, 11].

Treatment adherence is very important for the management of patients with low-back and neck pain. Despite the importance of this issue, due to the long course of treatment and the low self-confidence of patients in doing exercises, many patients stop continuing treatment and doing exercises [12, 13]. For this reason, the first and most important step in the management of these two disorders is the continuation and adherence to the treatment along with the participation strategy and correct exercise training, which has been approved in most clinical guidelines [14]. Currently, exercise therapy has been introduced as one of the most important treatment methods to reduce disability and improve chronic back and neck pain. It reduces the risk of dangerous diseases and increases life expectancy [15, 16].

On the other hand, in the management of low-back and neck pain, measuring the clinical effectiveness and evaluating the severity of their pain is a necessity. There are many reliable tools for this, which can be used to determine the severity of the patient's pain and the success rate of the treatment plan [17, 18]. Choosing an outcome measure that is easily scored and provides an objective measure to evaluate the severity of pain is essential in the process of managing low-back and neck pain [19, 20].

Rehabilitative treatment approaches benefiting from e-health have created a tremendous opportunity to improve patient care and disease management [21]. Electronic health solutions have contributed to the individual's participation in self-care, increased treatment efficiency, and adherence to treatment methods. The mobile application in the field of physiotherapy and rehabilitation causes the following: (a) increasing patients' access to healthcare and health-related information, (b) improving the ability of physiotherapists to diagnose and track neurological and muscular diseases and expand access to online education, and (c) self-management in physiotherapy treatment, which these three factors have facilitated physiotherapy treatment methods [22, 23]. Despite a large number of applications in the field of low-back and neck pain management and numerous articles evaluating them [24–26], the lack of an application with an agreed conceptual and content model is a clear information gap. Also, most of the applications in the field of physiotherapy for low-back and neck pain do not use clinical guidelines and valid references for their content [24–27]. For this reason, patients and therapists face challenges in choosing the appropriate evidence-based application [28].

Preparing the conceptual and content model of applications from guidelines and reliable sources is in the direction of evidence-based medical policies as it is one of the most important steps in the design of any information system. In various fields of medicine, we are slowly growing evidence-based development of technologies. For example, Nadri et al. introduced a mobile app for self-care as a complementary approach for cutaneous leishmaniasis. This application provides the best necessary treatment approaches according to user data and based on evidence-based information [29]. Also, Ehrler et al., to design a mobile app for bedside nursing care, invited 11 participants to help them choose the most important features to be integrated into the tool with brainstorming sessions [30].

Considering that the desired goals cannot be achieved with the existing applications, this study tries to meet this need with evidence-based content and validation of this content. Therefore, the present study is aimed at developing a conceptual and content model of a mobile-based application for the management of people with low-back and neck pain.

## 2. Materials and Methods

**Figure 1:**
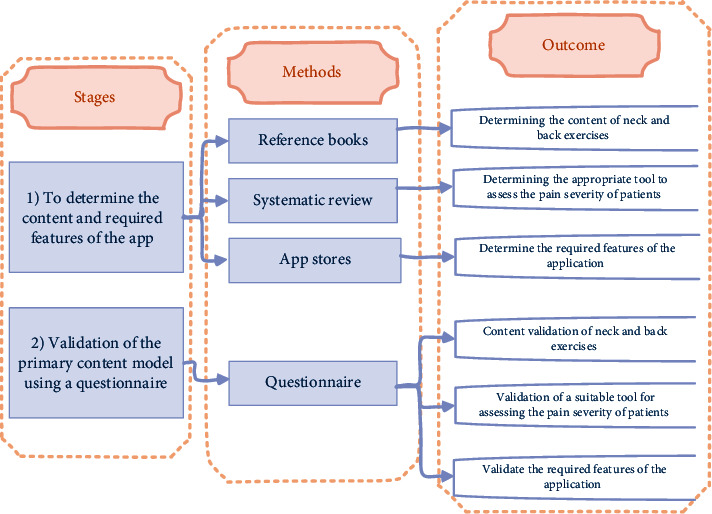
Executive stages and details of study implementation.

This is a descriptive-analytical study, which was conducted in 2022 as a combination of quantitative and qualitative methods. The study was conducted in two separate stages, which include (1) determining the content and required features and (2) validating the initial content model.  Figure 1 shows the details of the stages.

### 2.1. First Stage: Determining the Content and Required Features

During the brainstorming sessions, the research team considered three separate steps for this stage.

1. Determining the content of low-back and neck exercises
2. Determining the appropriate tool to assess the pain intensity of patients
3. Determining the required features of the application

In the following, the research method is explained in detail in each of these parts.

Due to the need to use standard and reliable sources whose validity has been confirmed, the research team decided to use reference books to determine low-back and neck exercises and educational materials.

The second part was related to determining the appropriate tool to assess the severity of patients with low-back and neck pain. Using these tools, patients can assess the severity of their pain and current condition. To find the best tool to use in the application, we tried to extract all the tools available in the assessment of pain intensity using a systematic review. A systematic search was performed using the PRISMA guidelines to retrieve all available studies. Scopus, PubMed, Web of Science, and ScienceDirect were searched along with Google Scholar search engines to extract the valid tools available. The search was performed with the keywords “endpoint”, “physical therapy”, “Neck Pain”, “Low Back Pain”, “Pain Management”, “pain intensity”, and “effect measure”. The inclusion criteria included studies on back or neck pain instruments in English without time limits, and the exclusion criteria included general pain management scales. In the end, the features of these tools were compared to choose the best scale for use in the content model.

In the third step from the first stage, to determine the required features of the low-back and neck pain application, the research team used the reviewing of existing applications. With this aim, the common features between the apps and those defects were identified. This makes it possible to design a new app by understanding the existing conditions and deficiencies. To identify these features, the Google Play Store was searched based on the keywords “low back pain”, “low back pain”, “lumbago”, “neckache”, and “neck pains”. The applications were reviewed according to the inclusion and exclusion criteria, and eligible cases were included in the study. The inclusion criteria included availability to the public, and no need for an accessory device to perform interventions, updated in 2019 to 2021, providing an exercise solution to encourage the patient to perform daily activities. Exclusion criteria also include providing anatomical, preventive, and diagnostic information without exercise, personalized applications for a specific condition such as pregnant women and people with sciatica pain and back or neck pain due to cancer, apps containing software errors, and apps that provide yoga and relaxation therapy solutions. Eligible apps were installed on mobile phones and evaluated by two members of the research team with the Mobile Application Rating Scale (MARS) questionnaire, and the results were recorded in MS Excel 2019. The results of this section are presented in detail in a separate article and are currently under review in the Journal of Healthcare Engineering.

### 2.2. Second Stage: Validation of the Basic Content Model

After the first stage, a list of the basic content of the app was extracted in all three sections. This was considered a basic content model for the application. To validate and get expert opinions, this model was converted into a questionnaire. The questionnaire included three parts: (1) demographic information of experts, (2) the basic content model of the app, and (3) open questions about the content model. The second part included 40 data elements with three axes of low-back and neck exercises (11 elements), pain intensity assessment tool (10 elements), and required characteristics of the app (19 elements). Each question in the questionnaire has five options based on a 5-point Likert scale: “completely agree =5,” “agree =4,” “I have no opinion=3,” “disagree =2,” and “completely disagree =1.” To determine validity and reliability, it was given to 10 people specializing in physiotherapy and medical informatics. After collecting the questionnaires and analyzing the results, content validity and reliability were obtained as content validity ratio (CVR) = 0.62 and Cronbach's alpha (*α* = 0.66). Content validity assesses whether an instrument covers all relevant aspects of the topic that it is intended to measure. To obtain CVR, experts are asked to determine whether an item is necessary to implement a construct in a set of items or not. A higher score indicates greater panel members' agreement on the necessity of an item in the instrument. Cronbach's alpha is also a method to evaluate reliability by comparing the amount of common variance or covariance among the items that make up an instrument with the amount of overall variance. After determining the validity and reliability, the questionnaire was made available to 15 physiotherapists in medical science universities in Iran and sports medicine specialists in the Sports Medicine Research Center of Tehran University of Medical Sciences by convenience sampling method. The criteria for entering this study were based on user responsiveness, expertise, familiarity with research, and interest in cooperation. 12 specialists (11 physiotherapists and 1 sports medicine specialist) collaborated with the research team. After collecting the questionnaires, SPSS software version 26 was used for data analysis. Data elements in all axes were analyzed based on descriptive statistics indicators such as minimum and maximum score, mean, percentage of content validity ratio, and the degree of interrater reliability.

## 3. Result

### 3.1. Determining the Content and Required Features of the Application

The results of this stage are shown in three separate parts.

#### 3.1.1. First Step: Determining the Content Model of Back and Neck Exercises

The content of exercises and how to display them were considered by the research team and reference books. The main part of the content was determined based on the book *Low Back and Neck Pain: Causes and Conservative Treatment* authored by Paul Williams [31]. This book is a resource used to teach the management of low-back and neck pain for undergraduate students of physiotherapy in the Faculty of Rehabilitation at Tehran University of Medical Sciences. In this book, essential exercises for prevention and conservative treatment are presented.  Table 1 shows the sports exercises selected by the research team from this book along with the description of each exercise.

#### 3.1.2. Second Part: Determining the Appropriate Tool to Assess the Pain Intensity

**Figure 2:**
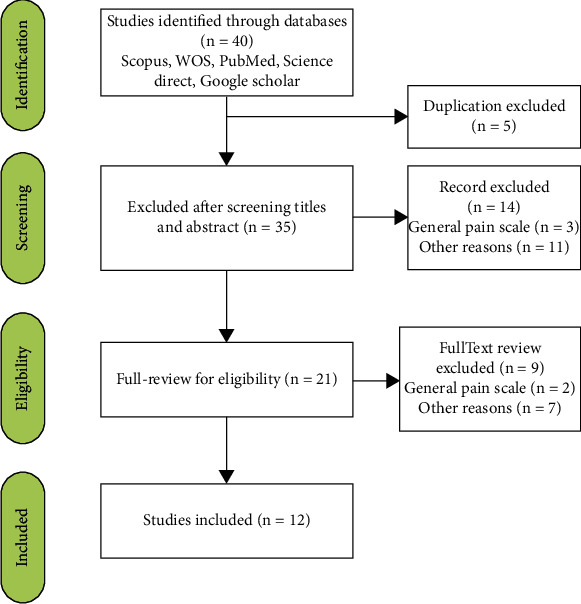
PRISMA flow diagram.

Pain intensity assessment tools were extracted using a systematic review of relevant studies. After extracting the studies, first, all duplicate and unrelated articles were removed from the study and only articles that met the inclusion criteria were left. In summary, the obtained results included 40 articles, and 12 pain intensity assessment tools were extracted from these articles. The PRISMA flow diagram shows the study review process (Figure 2).

After identifying the pain intensity assessment tools, the information on each of them was extracted and compared.  Table 2 provides comprehensive information on each of these tools.

#### 3.1.3. The Third Part: Determining the Required Features of the Application

After examining the apps available in Google Play, 234 neck pain and 246 back pain apps were identified in the initial phase. Of these, 14 apps (8 back pain and 6 neck pain apps) were included in the study based on the inclusion and exclusion criteria. To identify the required features of the application, the content and features of each of the included apps were reviewed. Also, to evaluate more accurately and measure their quality, the evaluation of these programs was done with the MARS questionnaire. Then, the strengths and weaknesses of the apps available in Google Play were extracted. Finally, a series of features were obtained as required features of neck pain and back pain application. The results of this section are shown in Table 3.

In Table 3, criteria 1 to 3 of the MARS questionnaire examine the presence or absence of desired features in the apps, and criteria 4 to 6 evaluate the quality of the apps. The scores of criteria 4-6 are also obtained from the average scores of two reviewers. The results of the Google Play app reviews showed that there were some essential information elements in some apps, but none of them were evidence-based. However, the “Low back pain relief exercises at home” application with a score of 3.79 scored a higher average score among other applications in terms of compliance with the MARS criteria. Among the neck pain apps, the highest score of 3.58 was related to the Neck & Shoulder Workout (30 days Workout Plan) app. In this app, for 30 days of the month, exclusive sports are designed with animation. However, it should improve aspects such as evidence-based and information quality to increase its impact on users and ensure greater security and privacy.

### 3.2. Findings Related to the Validation of the Content Model

The basic content collected in the first stage was provided to the experts in the form of a questionnaire for validation.  Table 4 shows the demographic information of the participants at this step. In this survey, most participants were women (66.67 percent), the highest frequency related to the academic rank of assistant professor (41.67 percent), and most participants were from the Tehran University of Medical Sciences (50 percent).

The survey results are shown in Table 5 which is the final content model created in this study. Out of 40 data elements proposed in the questionnaire, 33 elements are considered essential by experts (Table 5). All data elements based on exercises and pain intensity assessment scale (CVR > 0.62) remain in the study. Instead, in the axis of the app's features, out of 19 data elements, 7 elements including electronic communication, medication reminder, mental activity tool, physical activity planning, motivational notes, recording the time of three meals, and finding the nearest care centers were excluded, and 12 elements remained.

Also, the axis of the FRI pain intensity assessment scale attracted favorable opinions with an average score of 100% (CVR = 1). This axis has the highest score among the three axes. The FRI was developed by Feise and Menke in 2001, and its validity and reliability have been confirmed in several languages. It is a 5-point Likert scale from 0 (no pain) to 4 (worst possible pain) [43, 44]. The Persian version of this scale was translated by Dr. Ansari et al., in 2012, which has sufficient validity and reliability [42]. The reason for choosing the FRI scale among all pain intensity assessment scales was that the research team chose this scale because of its ease and the need for less time to complete, as well as the existence of various versions in different languages, especially the Persian version.

## 4. Discussion

According to the findings, the minimum necessary data elements for the content model of the low-back and neck pain management app are 33 elements, which are determined in the 3 axes of exercises, pain intensity assessment scale, and app features. The results showed that the data elements of the content model, except for some features of the application, were agreed upon by the majority of experts.

### 4.1. Data Elements of Content Model Based on Exercises

The data elements of this axis include 8 exercises for back pain and 3 for neck pain, which are best used as animations in the app. Text instructions, audio, and video files are also recommended for each of the exercises for more clarity on how to do them correctly in the content model. Physiotherapy is usually part of the treatment provided by physical therapists to back pain or neck pain patients. In the current study, 8 exercises for the back and 3 exercises for the neck were determined.

Dutch clinical guidelines consider patient education and exercise therapy as the main contributions of physiotherapists in the treatment of back pain patients [45]. It also showed that physiotherapy compared to other approaches (such as hot packs, massage, stretching, mobilization, short-wave application, ultrasound, stretching exercises, manual therapy exercises, and electrotherapy) becomes more effective [45]. The clinical guidelines of the American Medical Association and the American Pain Society for the noninvasive treatment of acute, subacute, and chronic low-back pain have recommended physiotherapy according to the patient's condition [46].

In the clinical guidelines for neck pain of the American Physical Therapy Association, exercise and patient education are recommended [47]. The recommendations of the guidelines confirm the findings of the current study on physiotherapy data [44, 47]. Also, various studies have investigated the effectiveness of various web-based systems for performing physiotherapy exercise programs at home and the use of apps to train patients, which have had positive results [48, 49].

In this study, the data elements of exercises are included in the content model in the form of animation to perform correct movements. The experts participating in the validation considered the animated exercises to be effective. They also suggested the use of more exercises in a personalized way for each patient.

### 4.2. Data Elements of Pain Intensity Assessment Scale

The data elements of this axis were selected based on the FRI scale. It is useful for evaluating pain intensity and can report a patient's condition [43, 44]. Currently, pain intensity measures are widely used as valuable tools for researchers, physicians, patients, and payers. In the study of Foroutani et al., the responsiveness of the Persian version of the Functional Rating Index in patients with chronic nonspecific neck pain was investigated. This study showed that this scale has a good response and has practical importance for use in the clinic and research [50].

The importance of self-assessment of the health status of the patient or the use of patient-reported outcome measures (PROMs) and reporting provides a valid and reliable justification for the treatment of the patient. The results of measures are also used to analyze the quality of care. Also, with the increasing patients' participation in the care, the use of measures based on the patient's perspective has become increasingly complementary to clinical methods. The results of the use of PROMs in various studies have shown that these tools are essential to demonstrate the value and success of physiotherapy and are an essential tool [17, 18].

### 4.3. Data Elements of Application Features

The elements of this axis were collected by comparing the evaluation results of Google Play apps and were validated by experts. Based on the identification of gaps and strengths, a set of primary data elements was extracted, and finally, the validation results showed that out of 19 elements, only 12 elements with a CVR greater than 0.62 were recognized as essential. The importance of using animation in physiotherapy in a study by Zernicke et al. showed that the use of exercise programs using game consoles for patients with rheumatoid arthritis had similar effects compared to standard exercises at home, so such a program can be an alternative support option for patients with rheumatism [51].

Chiensriwimol et al. investigated the effectiveness of an exercise simulator for the treatment of a frozen shoulder in a mobile application. The study showed that the use of animation to simulate arm movements in different types of exercises using biofeedback data is effective for the treatment process and physiotherapy because they can remotely monitor and manage the patient's rehabilitation process [52]. In Chandra et al.'s study, health technology was used to reduce the difficulties of performing physiotherapy at home and reduce recovery periods. They used a muscle activity sensor connected to a mobile phone to increase the adaptation of sports movements. They argued that health technologies have many capabilities to offer their users, but they should be designed to match the lifestyle and real needs of patients [53].

On the other hand, the importance of patient education as an independent intervention or together with other interventions for people with musculoskeletal pain has been emphasized in several studies. The study of Goff et al. investigated the effect of training patients with knee arthritis in improving pain and function. The results showed that patient education is not an independent treatment and should be combined with physiotherapy to be more effective in statistics and clinical performance than education alone [54]. The results of Ramos-Remus et al.'s study on the education of rheumatic patients showed that patient education is one of the ways to achieve quality improvement in this disease. It is also emphasized that education is not only a program, but it is a strategy [55]. The results of the current study also emphasize the importance of using educational videos for conservative and preventive treatment and animations for correct exercises. This is consistent with the results presented in similar articles and promotes education as a special strategy alongside physiotherapy.

The importance of using reminders to prevent patients from losing motivation to exercise has been investigated in several studies. Jangi et al. conducted a systematic review titled the effect of reminders in physiotherapy, and the results showed that 35% of the studies reported positive effects of reminders [56]. They also concluded that the use of reminders is a useful strategy to improve patients' adherence to exercise programs [56]. This study was consistent with current research and showed that the development of technology and communication offers new ways to increase patient motivation. In the data elements of this content model, there is the use of warning and note setting to perform exercises, which was approved by experts with 100% votes as an essential element.

The importance of privacy and security of health data is considered sensitive by nature and according to the law, and therefore, it is very important to protect them. As the new capabilities of mobile phones are enhanced and allow millions of applications to take advantage of vast amounts of data, the importance of protecting health data becomes even more important [57]. Papageorgiou et al. analyzed the security and privacy of health applications, which showed that most applications do not take into account well-known privacy practices and guidelines, which puts the security of health users' data at risk [57]. Also, in another study conducted by Martínez-Pérez et al. to evaluate the privacy of health applications, it was shown that special protection of users' personal health information is important. However, the appropriate methods for doing this are not considered by app designers, and insecure applications are published [58]. In this study, to preserve the privacy of patients in the content model, authentication and other security aspects such as access control have been used, which received 100% of experts' favorable opinions.

Using health information technology and mobile applications to complete the rehabilitation of low-back and neck pain, which includes physiotherapy, home exercise, and patient education, is effective [59]. Due to the lack of monitoring of low-back pain and neck pain applications, the current study is the first essential step to determine an evidence-based content model with 33 data elements to increase the motivation and adherence of patients to perform correct exercises. Treatment is by mobile health application. Among the advantages of this study are the use of reliable sources to extract the content of the application, the use of special features of the applications, attention to the gaps to solve them, and the validation of the content model by experts.

## 5. Limitations

The main limitation of this research is the number of exercises, which should be increased based on acute, subacute, and chronic low-back pain and also based on types of neck pain. Also, exercises should be implemented for each patient in a personalized way. Another limitation was the use of guidelines and articles in English and Persian and not using other languages.

## 6. Conclusions

According to the findings, a content model was presented in 3 axes. The purpose of all the different axes is to increase the patient's willingness to do exercises and the correct way to perform exercises and conservative treatment and check the progress of the treatment. It is possible to improve the course of treatment by providing the possibility of home care performing exercises in a principled manner and making the patient adhere to the continuation of the treatment. It is also possible to improve their communication with the providers through the reports sent by the patient and enable the analysis of the effectiveness of exercises. These goals are possible through approved animated exercises along with instructions on how to perform them, standard pain intensity assessment, and the use of mobile application features. Also, considering the identified gaps in Google Play applications, determining the content model of the application that is based on evidence and validated by experts is useful in improving apps in this field. Developers can use these findings as a basis for designing new applications to manage lower back pain and neck pain.

## Figures and Tables

**Table 1:** Content of low-back and neck pain exercises.

Num	Exercise picture	Description	Num	Exercise picture	Description
Low-back pain exercises					
1	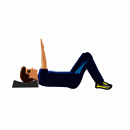	Strengthen the less-used abdominal muscles	2	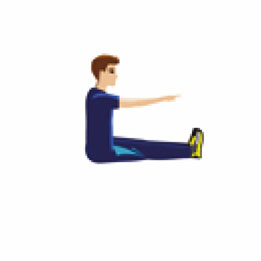	Simultaneous stretching of muscles and nerves and painless walking and strong and short stretching of the low-back muscles and improving the range of motion towards the front of the waist
3	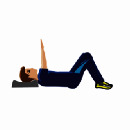	Strong contraction of the abdominal muscles	4	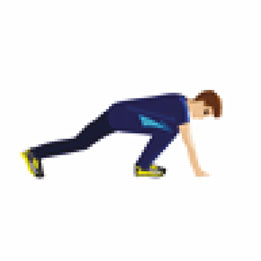	Stretch on the bar and increase the forward range of motion of the pelvis. Reduce the deflection of the front of the pelvic plate
5	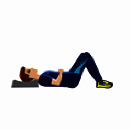	Strengthen the weak serine muscles (a group of three muscles that make up the buttocks)	6	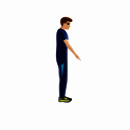	Strengthen the muscles of the legs with special emphasis on the muscles of the front of the thighs (quadriceps muscles) and strengthen the muscles (buttocks) of the serine
7	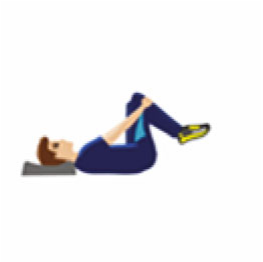	Strong and short low-back muscle stretch and improve the forward range of motion of the waist	8	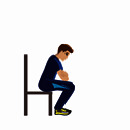	Strengthen leg muscles with special emphasis on front thigh muscles (quadriceps muscles) and strengthen serine muscles

Neck pain exercises					
1	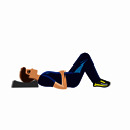	Strengthen the neck muscles	2	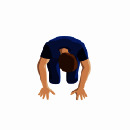	Strengthen the neck muscles
3	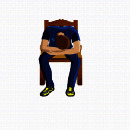	Strengthen the neck muscles			

**Table 2:** Comparing pain intensity indexes for low-back pain and neck pain.

Num	Tool name	Developer	Goal	Items	Score method	Description
1	Quebec Low Back Pain Disability Scale (QBPDS) [32]	Kopec et al. [32]	Considering functional limitations related to pain, monitoring patients' progress, and comparing the evolution of LBP individuals in rehabilitation programs	20 daily activities	Scoring from 0 means “performing the activity without problems” and 10 means “unable to do”	Patients should choose the score that best describes their current level of ability in each activity
2	Oswestry Disability Index (ODI) [33]	Fairbank et al. [33]	The target population of this scale is people who suffer from acute back pain. This scale is used by clinicians and researchers to quantify back pain disability	10 daily activities	They are rated from 0 “I have no pain” to 5 “The pain is the worst possible”	It is most effective for persistent severe disability and is published in at least four formats in English and nine languages
3	Roland-Morris Disability Questionnaire (RMDQ) [19]	Roland-Morrisn et al. [34]	The target population of this scale is very suitable for patients with acute, subacute, or chronic low-back pain with mild to moderate pain	Scale of 24, 18, and 11 questions	Average scores	This scale is best suited for mild to moderate back pain disability and can be completed in person, electronically, or over the phone
4	Chronic Pain Grade Questionnaire (CPGS) [35]	Von Korff et al. [35]	For use in all chronic pain conditions including chronic musculoskeletal disorders (MSK) and back pain	7 indicators	Based on numerical scores, which are scored from 0 “no pain” and 10 “unbearable pain”	This scale was designed before the International Classification of Functioning, Disability, and Health of the World Health Organization (ICF). However, recent studies have shown that this index measures all ICF criteria
5	Patient-Specific Functional (PSFS ([36]	Stratford et al. [36]	To provide clinicians with a valid, reliable, responsive, and efficient outcome measure for the management of back pain and neck pain, easy to use, and applicable to a large number of clinical presentations	11-point Likert indicators of five important activities	“0” indicates “unable to perform,” and “10” indicates “ability to perform at previous level”	It includes two stages before and after the intervention
6	P4 Screener [37]	Cole et al. [37]	Attempts were made to improve the “one-dimensional rating scale”	4 indicators (morning, afternoon, evening, and activity during the last 2 days)	Scores from 0 (no pain) to 10 (highest possible pain level)	Ability to complete in less than a minute and analyze in 5 seconds
7	Neck Pain Disability Index Questionnaire (NPAD) [38]	Goolkasian et al. [38]	This scale is specially prepared for patients with neck pain	20 indicators	Points based on numerical scoring	It is valid for assessing outcomes in patients with neck pain. It is easy to complete and grade
8	Copenhagen Neck Disability Scale (CNFDS) [39]	Jordan et al. [39]	A useful tool for self-care of patients with neck complaints treated with physiotherapy	Including questions related to headaches, ability to sleep, concentration, and activities of daily living, as well as questions of a psychosocial nature	Scoring as “yes,” “sometimes,” and “no”	Available for patients from 20 to 75 years
9	Northwick Park Questionnaire (NPQ) [40]	Northwick Park Hospital	Objective measurement for outcome assessment and symptom monitoring in patients with acute or chronic neck pain over time	The 9th five-part section and the 10th question are related to the comparison of the current status with the status of the last completion time	Score based on numerical scoring	Good reliability, high internal consistency, and sensitivity to change
10	Neck Disability Index (NDI) [20]	Vernon et al. [20]	Use to evaluate the condition of patients and the evolution process during treatment	10 six-part sections	Score based on numerical scoring	The target population includes people with chronic neck or upper back pain, radiculopathy, neck injuries, and thoracic disc syndrome
11	Bournemouth Questionnaire (BQ) [41]	Bolton [41]	The scale is based on ICF dimensions, which also pays attention to the emotional and cognitive aspects of neck pain and back pain. It is designed for patients with nonspecific back or neck pain	7 questions	Points from zero to 10 for each question	The first version is for measuring different dimensions of pain in patients with back pain, and the second version is for evaluating pain in patients with nonspecific neck pain
12	Functional Rating Index (FRI) [42, 43]	Feise and Menke [43]	To assess the condition of the neck, chest, and back, which reduces the need for multiple scales for diseases of the spine	10 questions	Points from 0 to 4 for each question	Adequate validity and reliability, requiring only one minute to complete and approximately 20 seconds for clinician scoring

**Table 3:** Assessment 14 specifically chosen Google Play apps by MARS.

Num	MARS measurement criteria	Index	Back pain apps								Neck pain apps					
			Back pain relief exercises at home	Back pain exercises 2	6-minute back pain relief	Low-back pain relief exercises	Lower back pain exercises	Back pain relief exercises	Lower back pain and sciatica relief exercises	Back pain relief exercises at home	Relieve neck pain	My neck	Neck pain relief exercises	Neck stretches and exercises	Neck and shoulder pain relief exercises and stretches	Neck and shoulder workout
1	App targets	Increase happiness/well-being	—	Y	—	—	—	Y	—	Y	—	—	Y	Y	Y	Y
		Reduce negative emotions	—	—	—	—	—	Y	—	Y	—	—	—	—	Y	Y
		Depression anxiety/stress	—	—	—	—	—	—	—	—	—	—	—	—	—	—
		Anger	—	—	—	—	—	—	—	—	Y	—	—	—	—	—
		Behavior change	—	—	—	—	—	Y	—	Y	—	—	—	—	—	Y
		Alcohol/substance use	—	—	—	—	—		—		—	—	—	—	—	
		Goal setting	—	Y	—	—	—	Y	—	Y	—	—	—	—	Y	Y
		Entertainment	Y	Y	Y	Y	—	Y	—	Y	—	Y	Y	—	Y	Y
		Relationships	Y	Y	Y	Y	Y	Y	Y	Y	Y	Y	Y	Y	Y	Y
		Physical health	Y	Y	Y	Y	Y	Y	Y	Y	Y	Y	Y	Y	Y	Y

2	App theoretical background/strategies	Assessment	—	Y	—	—	—	Y	—	Y	—	Y	Y	Y	—	Y
		Feedback	—	Y	Y	—	—	Y	—	Y	Y	Y	Y	—	—	Y
		Information	Y	Y	Y	Y	Y	Y	Y	Y	Y	—	Y	Y	Y	Y
		Education	—	—	—	—	—	—	—	—	—	—	—	—	Y	—
		Monitoring tracking	—	—	—	—	—	—	Y		—	—	—	—	Y	—
		Goal setting	—	Y	—	—	—	Y	—	Y	—	—	Y	Y	—	Y
		Advice, tip, strategy, and skill training	—	Y	—	—	—	Y	—	Y	Y	—	Y	Y	—	Y
		Mindfulness	—	—	—	—	—	—	—	—	—	—	—	—	—	—
		Meditation	—	Y	—	—	—	Y	—	Y	—	—		Y	Y	Y
		Relaxation	—	Y	—	—	—	Y	—	Y	—	—	—	—	—	Y
		Gratitude strength-based	—	Y	Y	—	—	Y	Y	Y	Y	—	Y	Y	Y	Y

3	Technical aspects of the app	Allows sharing (Facebook and Twitter)	Y	Y	Y	—	Y	Y	Y	Y		—	Y	—	Y	Y
		Has an app community	—	—	—	—	—	—	—	—	—	—	—	—	—	—
		Allows password protection	—	—	—	—	—	—	—	—	—	—	—	—	—	—
		Requires login	—	—	—	—	—	—	—	—	—	—	—	—	—	—
		Sends reminders	—	—	—	—	—	—	—	—	—	—	—	—	—	—
		Needs web access to function	—	—	Y	—	—	Y	Y	Y	Y	Y	Y	—	Y	Y

4	App quality mean score (max score = 5)	Engagement	2.50	2.90	2.50	2.10	1.40	3.80	1.50	3.90	2.50	2.20	2.90	3.10	2.50	3.10
		Functionality	3.38	3.50	3.50	3.00	1.50	3.75	3.00	4.00	3.25	3.38	3.38	3.50	3.50	3.88
		Aesthetics	1.84	1.88	3.00	1.75	1.13	3.44	1.63	3.83	2.17	2.17	3.17	3.00	3.67	3.67
		Information	2.50	2.10	3.00	2.00	2.00	3.44	1.90	3.43	2.75	2.25	2.92	2.92	3.50	3.67

5	App subjective quality (max score = 20)	Deal with the user experience with the app questions	11	13.5	11	10.5	7.5	14.5	9.5	18	12.5	12	13	12.5	13	17

6	APP-specific	The impact of the app on people's attitudes	19.5	18.5	20	16	12.5	24	11.5	25.5	20	22	24	21.5	18.5	24

**Table 4:** Demographic distribution of participation.

Demographic characteristics		Frequency	Percentage
Gender	Male	4	33/33
	Female	8	66/67
	Sum	12	100

Organizational affiliation	Tehran University of Medical Sciences	6	50
	Shahid Beheshti University of Medical Sciences	2	16.6
	Rasht University of Medical Sciences	1	8.3
	Hamedan University of Medical Sciences	1	8.3
	Iran University of Medical Sciences	1	8.3
	Sports and Exercise Medicine Research Center	1	8.3
	Sum	12	100

Academic rank	Assistant professor	5	41/6
	Associate professor	2	16/6
	Professor	2	16/6
	Physiotherapy specialist	1	8/3
	Fellowship	1	8/3
	Sports medicine specialist	1	8/3
	Sum	12	100

**Table 5:** The final content model created in this study by 33 data elements.

Axis name	Number	Main data elements	Mean^∗^	CVR^∗∗^
Exercise-based book	1	Low-back pain exercise 1	4/83	0/83
	2	Low-back pain exercise 2	4/83	0/83
	3	Low-back pain exercise 3	4/83	0/67
	4	Low-back pain exercise 4	4/92	0/67
	5	Low-back pain exercise 5	4/92	0/67
	6	Low-back pain exercise 6	4/92	0/67
	7	Low-back pain exercise 7	4/92	0/67
	8	Low-back pain exercise 8	4/92	0/67
	9	Neck pain exercise 1	4/92	0/67
	10	Neck pain exercise 2	4/92	0/67
	11	Neck pain exercise 3	4/92	0/67
	Total		4/89	0/79

Pain intensity rating based on FRI	1	Pain intensity	5	1
	2	Sleeping	5	1
	3	Personal care (washing, dressing, etc.)	5	1
	4	Travel (driving, etc.)	5	1
	5	Work	5	1
	6	Recreation	5	1
	7	Frequency of pain	5	1
	8	Lifting	5	1
	9	Walking	5	1
	10	Standing	5	1
	Total		5	1

Application capability-based app review	1	The daily exercise section and the exercises prescribed by the physiotherapist	5	1
	2	Use animation to show exercises	5	1
	3	Using an audio file to explain how to do exercises	5	1
	4	Playing background music while doing exercises	4/83	0/66
	5	Display the number of exercises performed in the application	4/75	0/83
	6	Increase or decrease the number of exercises	4/83	0/66
	7	Training for back pain and neck pain	5	1
	8	Using text, audio, and video for daily activities	5	1
	9	Pain intensity assessment with FRI scale	5	1
	10	Report section (number of exercises performed and FRI score)	4/92	0/83
	11	The ability to send warnings and reminders along with notes	5	1
	12	Access to the application with username and password	5	1
	Total		4/94	0/92

^∗^Mean: average responses of people regarding the necessity of the item (number between 1 and 5). ^∗∗^CVR: the number of experts who declared the item important or very important (scores 4 and 5) divided by the total number of experts.

## Data Availability

The data used to support the findings of this study are available from the corresponding authors upon request.
